# Identifying patterns of immune related cells and genes in the peripheral blood of acute myocardial infarction patients using a small cohort

**DOI:** 10.1186/s12967-022-03517-1

**Published:** 2022-07-21

**Authors:** Peng-Fei Zheng, Qiong-Chao Zou, Lu-Zhu Chen, Peng Liu, Zheng-Yu Liu, Hong-Wei Pan

**Affiliations:** 1 Cardiology Department, Hunan Provincial People’s Hospital, No.61 West Jiefang Road, Furong District, Changsha, 410000 Hunan China; 2 Clinical Research Center for Heart Failure in Hunan Province, No.61 West Jiefang Road, Furong District, Changsha, 410000 Hunan China; 3 Institute of Cardiovascular Epidemiology, Hunan Provincial People’s Hospital, No.61 West Jiefang Road, Furong District, Changsha, 410000 Hunan China; 4 Department of Cardiology, The Central Hospital of ShaoYang, No.36 QianYuan lane, Daxiang District, Shaoyang, 422000 Hunan China

**Keywords:** Weighted gene co-expression network analysis, Acute myocardial infarction, Immune cell subtype distribution pattern, Significant modules, Hub genes

## Abstract

**Background:**

The immune system plays a vital role in the pathophysiology of acute myocardial infarction (AMI). However, the exact immune related mechanism is still unclear. This research study aimed to identify key immune-related genes involved in AMI.

**Methods:**

CIBERSORT, a deconvolution algorithm, was used to determine the proportions of 22 subsets of immune cells in blood samples. The weighted gene co-expression network analysis (WGCNA) was used to identify key modules that are significantly associated with AMI. Then, CIBERSORT combined with WGCNA were used to identify key immune-modules. The protein–protein interaction (PPI) network was constructed and Molecular Complex Detection (MCODE) combined with cytoHubba plugins were used to identify key immune-related genes that may play an important role in the occurrence and progression of AMI.

**Results:**

The CIBERSORT results suggested that there was a decrease in the infiltration of CD8 + T cells, gamma delta (γδ) T cells, and resting mast cells, along with an increase in the infiltration of neutrophils and M0 macrophages in AMI patients. Then, two modules (midnightblue and lightyellow) that were significantly correlated with AMI were identified, and the salmon module was found to be significantly associated with memory B cells. Gene enrichment analysis indicated that the 1,171 genes included in the salmon module are mainly involved in immune-related biological processes. MCODE analysis was used to identify four different MCODE complexes in the salmon module, while four hub genes (*EEF1B2*, *RAC2*, *SPI1*, and *ITGAM*) were found to be significantly correlated with AMI. The correlation analysis between the key genes and infiltrating immune cells showed that *SPI1* and *ITGAM* were positively associated with neutrophils and M0 macrophages, while they were negatively associated with CD8 + T cells, γδ T cells, regulatory T cells (Tregs), and resting mast cells. The RT-qPCR validation results found that the expression of the *ITGAM* and *SPI1* genes were significantly elevated in the AMI samples compared with the samples from healthy individuals, and the ROC curve analysis showed that *ITGAM* and *SPI1* had a high diagnostic efficiency for the recognition of AMI.

**Conclusions:**

Immune cell infiltration plays a crucial role in the occurrence and development of AMI. *ITGAM* and *SPI1* are key immune-related genes that are potential novel targets for the prevention and treatment of AMI.

**Supplementary Information:**

The online version contains supplementary material available at 10.1186/s12967-022-03517-1.

## Background

Coronary artery disease (CAD) is a common chronic heart disease worldwide. The accumulation of a large number of lipids under the intima of the coronary artery leads to the formation of atherosclerotic plaque, which gradually leads to the narrowing of the vascular lumen, finally resulting in impaired blood perfusion of the myocardium [1]. CAD usually presents with a variety of different symptoms, including ischemic cardiomyopathy, stable and unstable angina, acute myocardial infarction (AMI), and even sudden accidental death [2]. Emergency percutaneous coronary intervention (PCI) can quickly restore cardiac perfusion and makes a great contribution in improving the prognosis of AMI patients. Nevertheless, AMI has becomea main cause of hospitalization and mortality in patients, especially in China, and its incidence is increasing annually [3]. Previous studies have shown that AMI is a complex disease that is influenced by multiple factors, such as inflammation responses [4], immune mechanisms [5], hypertension, hyperglycaemia, smoking, obesity and dyslipidemia [6]. Accumulating evidence also shows that total cholesterol (TC) and low-density lipoprotein cholesterol (LDL-C) exert a synergistic effect on the immune inflammatory response, which can increase oxidative stress and vascular inflammation, leading to reduced bioavailability of nitric oxide (NO), and ultimately the formation of atherosclerotic plaque [7]. Several researches have indicated that atorvastatin therapy can effectively reduce levels of LDL-C, interleukin (IL)-1, tumour necrosis factor-alpha (TNF-α), C-reactive protein (CRP), and IL-6 in patients with high cholesterol, compared with dietary control alone [8]. At present, lipid-lowering therapy has become the cornerstone of drug therapy for CAD or AMI. We can effectively reduce the occurrence of major adverse cardiovascular events (MACEs) by downregulating LDL-C level. However, even if the level of LDL-C is reduced significantly, even until levels close to that at birth, MACEs cannot be completely eliminated. Immune inflammatory responses may partially account for this residual risk. Clear inflammatory intervention can be expected to effectively further improve the prognosis of patients, compared with only a reduction in LDL-C levels. Recently, Fernandez et al. provided the first overview of the human immune cell landscape during atherosclerosis and provided insights into the identity of immune cells that reside in the plaque and described their different activation states, which has opened the door for the study of atherosclerosis caused by autoimmune response [9]. Furthermore, Yang et al. suggested that the activation of signal cointegrator 1 complex subunit 2 (*ASCC2*), solute carrier family 25 member 37 (*SLC25A37*), and leucine rich repeat containing 18 (*LRRC18*), can be used as diagnostic markers of CAD, while immune cell infiltration plays a crucial role in the occurrence and development of CAD [10]. However, the pattern of immune cell infiltration in AMI has not been fully elucidated. Therefore, clarifying immune infiltration in AMI and identifying the key genes associated with immune cells may provide a novel perspective on the prevention and treatment of AMI.

Along with increased popularization and application of gene chip gene-chip sequencing technology, microarray analysis has become a practical and novel method of identifying susceptive genes correlated with AMI [11], thus helping clinicians gain a deeper understanding of the relationship between genes and disease [12, 13]. However, the sensitivity and reproducibility of microarray analysis based on differentially expressed genes may be limited [14, 15]. Weighted gene co-expression network analysis (WGCNA) is used increasingly widely to analyse a large number of gene expression data and is a powerful systematic biological approach to analyse network relationships and molecular mechanisms [16]. WGCNA is often used to identify co-expressed gene modules that are of specific biological significance and explore the association between gene modules and interesting sample characteristics [17].

For the past few years, an increased number of studies have indicated that immune cell infiltration may play a critical role in the pathogenesis and progression of CAD. Yang et al. have suggested that there is an increased in the infiltration of monocytes coupled with the decreased infiltration of CD8 + T cells in patients with CAD [10]. However, immune cell infiltration in AMI has not been fully elucidated. CIBERSORT, is an analysis tool that is used widely to explore the infiltration ratio of 22 immune cells in the samples based on the expression profiles of microarray data or RNA-seq data [18]. At present, a few studies have combined WGCNA with CIBERSORT to identify key immune related genes involved in AMI. Therefore, to meet this demand, in this study, CIBERSORT was used to calculate the proportions of 22 types of immune cells in AMI patients, while WGCNA was used to identify key modules that are significantly associated with AMI. Thereafter, the CIBERSORT results were combined with WGCNA to identify immune-related key modules and genes in patients with AMI to help elucidate the immune related molecular mechanism of AMI and lay the foundation for the development of immunomodulatory therapy for AMI.

## Materials and methods

### AMI microarray datasets

**Fig. 1:**
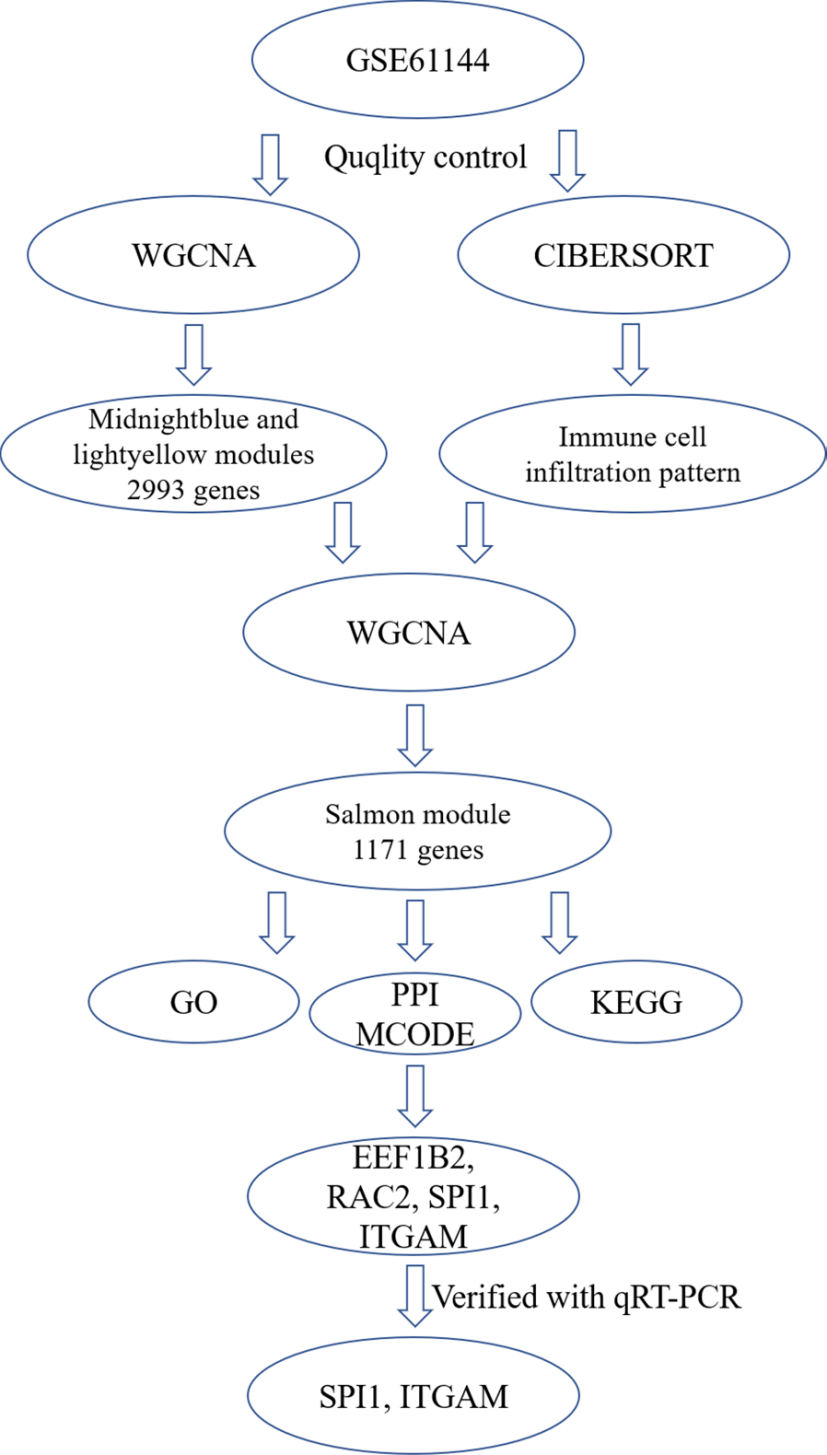
A flow chart for analysis. *GO* Gene Ontology annotation, *KEGG* Kyoto Encyclopedia of Genes and Genomes pathway enrichment analyses, *PPI* protein–protein interaction, *MCODE* molecular complex detection, *WGCNA* Weighted gene co-expression network analysis

The gene expression matrix of the GSE61144 dataset, which included ten normal and fourteen AMI samples was extracted from Gene Expression Omnibus (GEO, http://www.ncbi.nlm.nih.gov/geo) public database, which is based on the GPL6106 Illumina human-6 v2.0 expression beadchip platform. The ‘Normalize Between Arrays’ function of the *limma* package was used to normalize the gene expression matrix [19]. When a probe corresponded with multiple gene names, it was removed, and when multiple probes corresponded with the same gene, the average value of multiple probes was used as the true expression value of the gene. The specific workflow is shown in Fig. 1.

### WGCNA analysis identified modules that were significantly associated with AMI

As one of the most commonly used tools in systems biology, WGCNA can be used to construct a scale-free network based on gene expression data [20]. The genes with the top 25% of variance were selected for the WGCNA analysis. In this study, the appropriate soft threshold was defined as 18, and the WGCNA analysis was carried out according to methods detailed in our latest publication [21].

### Evaluation of immune cell subtype distribution and identification of modules significantly associated with immune cells

The CIBERSORT.R script was downloaded from the CIBERSORT website and was used to explore the immune infiltration pattern of AMI [18]. After the expression matrix of immune cells was obtained according to instructions given on the CIBERSORT website, the “ggplot2” software package was used to draw a histogram, heat map, and boxplot diagrams. The histogram showed the proportion of 22 immune cells infiltration in AMI patients, while the heat map and boxplot diagrams showed the difference in immune cell infiltration in control and AMI subjects. The "corrplot" software package in R software was used to calculate the Pearson correlation coefficient between each type of immune cells and display the results through the relevant heat map. Using the previously described method, the correlation between genes and immune cells was further explored based on the gene expression profiles of key modules to identify several novel key modules that were significantly associated with immune cells.

### Enrichment analysis of interesting modules

Kyoto Encyclopaedia of Genes and Genomes (KEGG) and Gene Ontology (GO) enrichment analysis of the genes in biologically significant modules was conducted using the clusterProfler and DOSE package in R [22]. The threshold was determined as *p*.adjust < 0.05.

### Identification of key genes and the correlation between key genes and immune cells

The Search Tool for the Retrieval of Interacting Genes (STRING) online database (version 11.0; http://www.string-db.org) was used to construct a protein–protein interaction (PPI) network based on genes in modules that were significantly associated with immune cells [23]. The PPI network was visualized using Cytoscape software [24]. The MCODE combined with cytoHubba plug-ins in Cytoscape software were used to identify hub genes. The Pearson correlation coefficient between the identified hub genes and each type of immune cell was calculated using the "corrplot" software package in R software and the results were visualized using a heat map.

### Study population

A total of 444 subjects with chest pain, which included 230 patients with AMI and 214 controls, collected from the Cardiovascular Department of Hunan Provincial People's Hospital. All cases suffering from AMI enrolled in this study received percutaneous coronary intervention (PCI) within 12 h after the onset of chest pain. AMI was diagnosed according to the 2018 diagnostic guidelines for AMI patients [25]: an electrocardiogram (ECG) showing new ischemic changes, echocardiogram indicating the loss of viable myocardium and/or new localized ventricular wall dyskinesia and serum levels of cardiac troponin T (cTnT) above the upper limit of 99% of the reference value. Sex- and age- matched healthy participants with no history of cardiovascular or other systemic diseases were also enrolled in this study based on ECG tests, blood, physical examination, and coronary angiography. Exclusion criteria are as follows: (1) active inflammation; (2) subjects treated with thrombolytic therapy and subjects suffering from cardiovascular and cerebrovascular diseases (such as cardiomyopathy, severe valvular abnormalities, atrial fibrillation, congenital heart disease or ischemic stroke); and (3) subjects with autoimmune diseases, tumours, renal and/or hepatic dysfunction. Laboratory findings, angiographic results and baseline clinical features of all subjects were collected and recorded in detail. Blood samples were obtained from AMI patients within hours of admission with an episode of chest pain and before an antiplatelet or anticoagulant was administered. The diagnostic criteria for hypertension and diabetes and the normal range of biochemical examinations were conducted as previously described [26, 27]. Study protocols were developed based on instructions from the Ethics Committee of Hunan Provincial People's Hospital and the 2008 revision of the Declaration of Helsinki of 1975 (http://www.wma.net/en/30publications/10policies/b3/). All subjects provided written and informed consent.

### RT-qPCR

Whole blood samples were obtained from all subjects and placed in a heparin vacuum tube for preservation. Subsequently, peripheral blood monocytes (PBMCs) were isolated using Ficoll‐Hypaque density gradient centrifugation by following the manufacturer's instructions. Total RNA was isolated from the isolated PBMCs using TRIzol reagent, according to the manufacturer’s instructions. cDNA was then reverse-transcribed using a PrimeScript RT reagent kit (Takara Bio, Japan). A Taq PCR Master Mix Kit (Takara) was used to perform the RT-qPCR based on an ABI Prism 7500 sequence-detection system (Applied Biosystems, USA). The proprietary of the qPCR primers used in this experiment were designed and validated by Songon Biotech (Songon Biotech, Shanghai, China). Statistical significance was considered to be indicated by a *p*-value < 0.05.

### Statistical analysis

SPSS (Version 22.0) was used for all statistical analyses in this study. Continuous data with a normal distribution between the AMI and normal groups were analysed using an independent sample *t*-test. Non normal distribution data, such as TG level, were expressed as median and quartile ranges, and were analysed using the Wilcoxon-Mann–Whitney test. The chi-square test was used to analyse measurement data, such as the number of drinkers and smokers, and the sex ratio. Based on previous studies [21], MedCalc software (MedCalc Software, Mariakerke, Belgium, version 19.7.4) was used to perform nonparametric receiver operating characteristic (ROC) curve analysis. R software (version 4.1.0) was used to perform the bioinformatics analysis. All tests were two-sided, and a *p* < 0.05 was considered to indicate statistical significance.

## Results

### Data pre-processing

The data were pre-processed by adding missing values, deleting outliers, and standardizing the data format. A total of 24,958 different gene symbols were screened in the 24 samples. The expression profiles of the 24,958 genes and the clinical features of the 24 samples are also shown in Additional file 2: Tables S1 and Additional file 3: Table S2.

### Weighted gene co‑expression networks

**Fig. 2:**
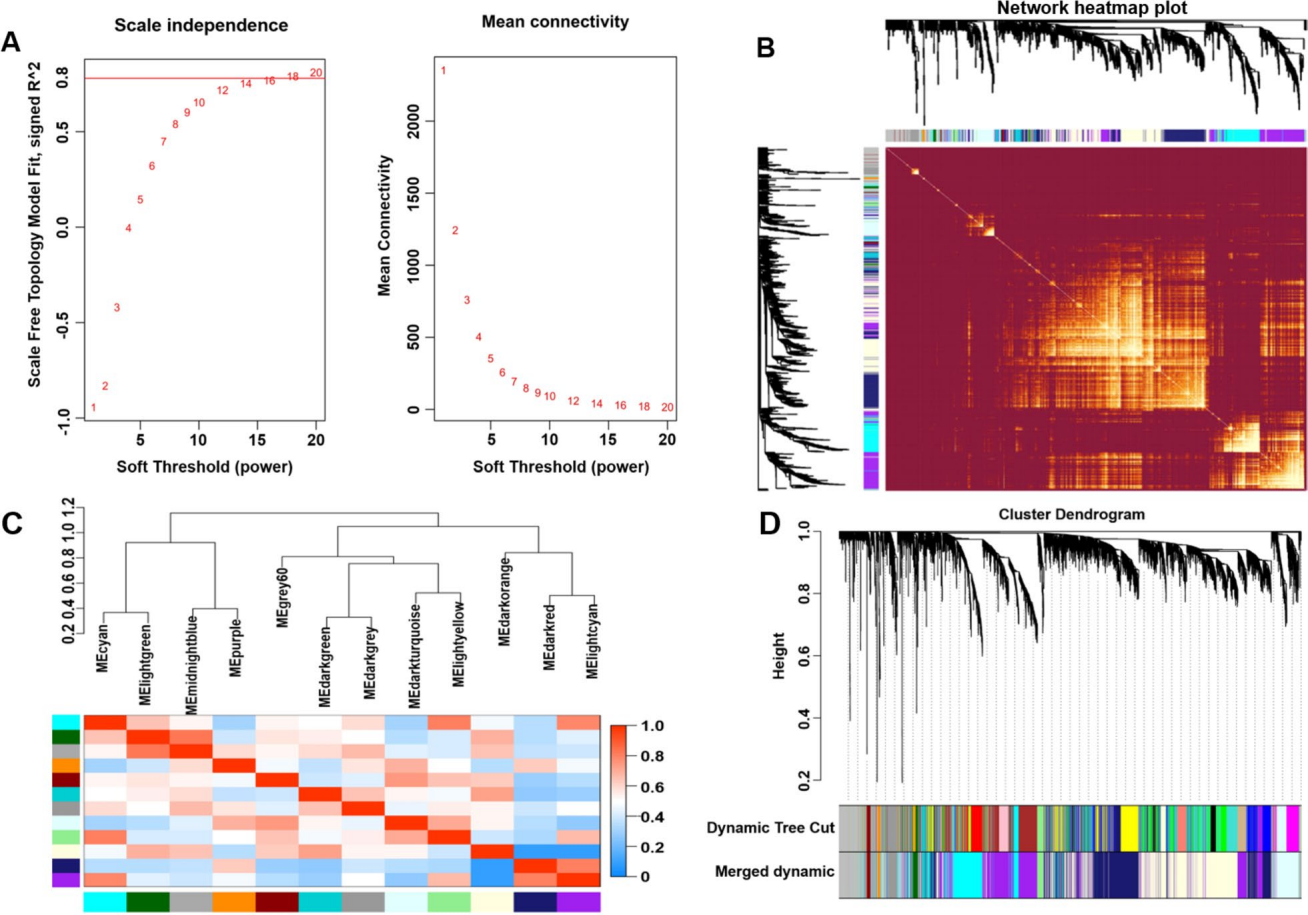
Weighted gene co-expression network analysis. **A** Analysis of network topology for various soft-thresholding powers. **B** Heatmap of the topological overlap in the gene network. **C** Relationship among all the modules. **D** Clustering dendrogram of genes. Gene clustering tree (dendrogram) obtained by hierarchical clustering of adjacency-based dissimilarity

After calculation, we found that when the correlation coefficient was greater than 0.8, the corresponding soft threshold was 18. Therefore, a soft threshold of 18 was selected to construct several gene modules (Fig. 2A). A topological overlap matrix was constructed by calculating the adjacency and correlation matrices of the gene expression profiles. Figure 2B depicts the gene cluster tree. Then, hierarchical mean linkage clustering combined with TOM were used to identify gene modules in each gene network. The heat map is shown in Fig. 2C. The dynamic tree cutting algorithm describes the 12 gene modules and is shown in Fig. 2D.

### Identification of the modules of interest

**Fig. 3:**
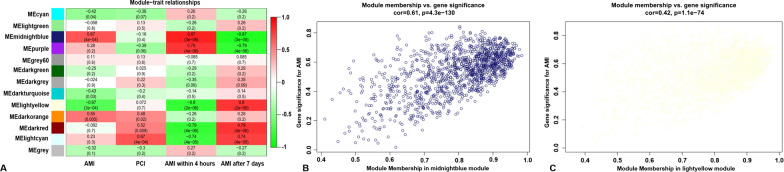
Module-feature associations. **A** Each row corresponds to a modulEigengene and the column to the clinical phenotype. Each cell contains the corresponding correlation in the first line and the *p*-value in the second line. The table is color-coded by correlation according to the color legend. Scatterplot shows a highly significant correlation between gene significant (GS) versus module membership (MM) with AMI in the midnightblue (**B**) and lightyellow (**C**) modules

Modules closely associated with disease characteristics are often found to maintain several specific and very important biological functions. As depicted in Fig. 3A, the midnightblue (*r *^*2*^ = 0.67, *p* = 4e-04) and lightyellow (*r *^*2*^ = -0.67, *p* = 3e-04) modules appeared to be highly correlated with AMI. Further in-depth calculations were performed to calculate the correlation coefficient between the colour module and gene significance. The correlation coefficient between the midnightblue module and gene significance was 0.61 (*p* = 4.3e-130) (Fig. 3B), while the correlation coefficient between the lightyellow module and gene significance was 0.42 (*p* = 1.1e-74) (Fig. 3C). A total of 2,993 gene symbols in the midnightblue and lightyellow modules and their GS values as well as corresponding *p* values are also shown in the Additional file 4: Tables S3.

### Profile of the immune cell subtype distribution pattern

**Fig. 4:**
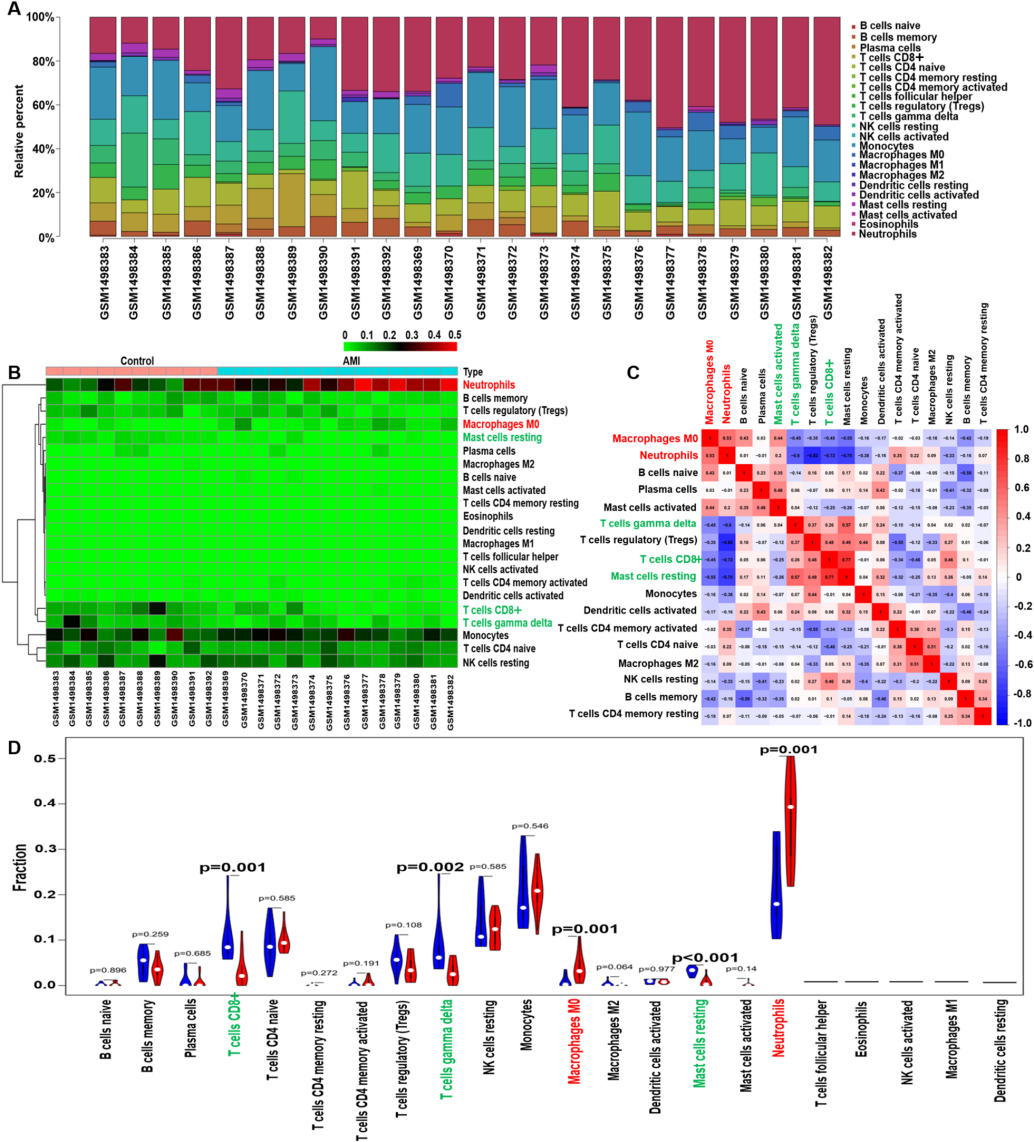
Infiltration pattern of immune cell subtypes in GSE61144 cohort. **A** The bar plot visualizing the relative percent of 22 immune cell in each sample. **B** Heatmap of the 22 immune cell proportions in each sample. **C** Correlation heatmap of all 22 immune cells. **D** Violin plot of all 22 immune cells differentially infiltrated fraction

The CIBERSORT algorithm was used to evaluate the differential expression of immune fractions between the control and AMI subjects. The cumulative histogram visually demonstrates the relative proportion of various immune cell subtypes (Fig. 4A). As shown in Fig. 4B, the heatmap showed that there were significant differences in the proportion of immune cells between the control and AMI samples. Using a correlation matrix, we found that neutrophils were positively correlated with M0 macrophages; and negatively correlated with Tregs, γδ T cells, CD8 + T cells, and resting mast cells (Fig. 4C). Compared with normal subjects, AMI samples generally had decreased infiltration of CD8 + T cells, resting mast cells, and γδ T cells, and increased infiltration of neutrophils and M0 macrophages (Fig. 4D). Due to the limitations of the CIBERSORT algorithm, the distribution of several immune cell subsets, including activated NK cells, follicular helper T cells (Tfhs), eosinophils, M1 macrophages, and resting dendritic cells, that have a low level of expression in AMI have not been fully elucidated. In addition, the immune cell infiltration pattern in AMI is also shown in Additional file 5: Tables S4.

### Identification of modules that are significantly associated with immune cells

**Fig. 5:**
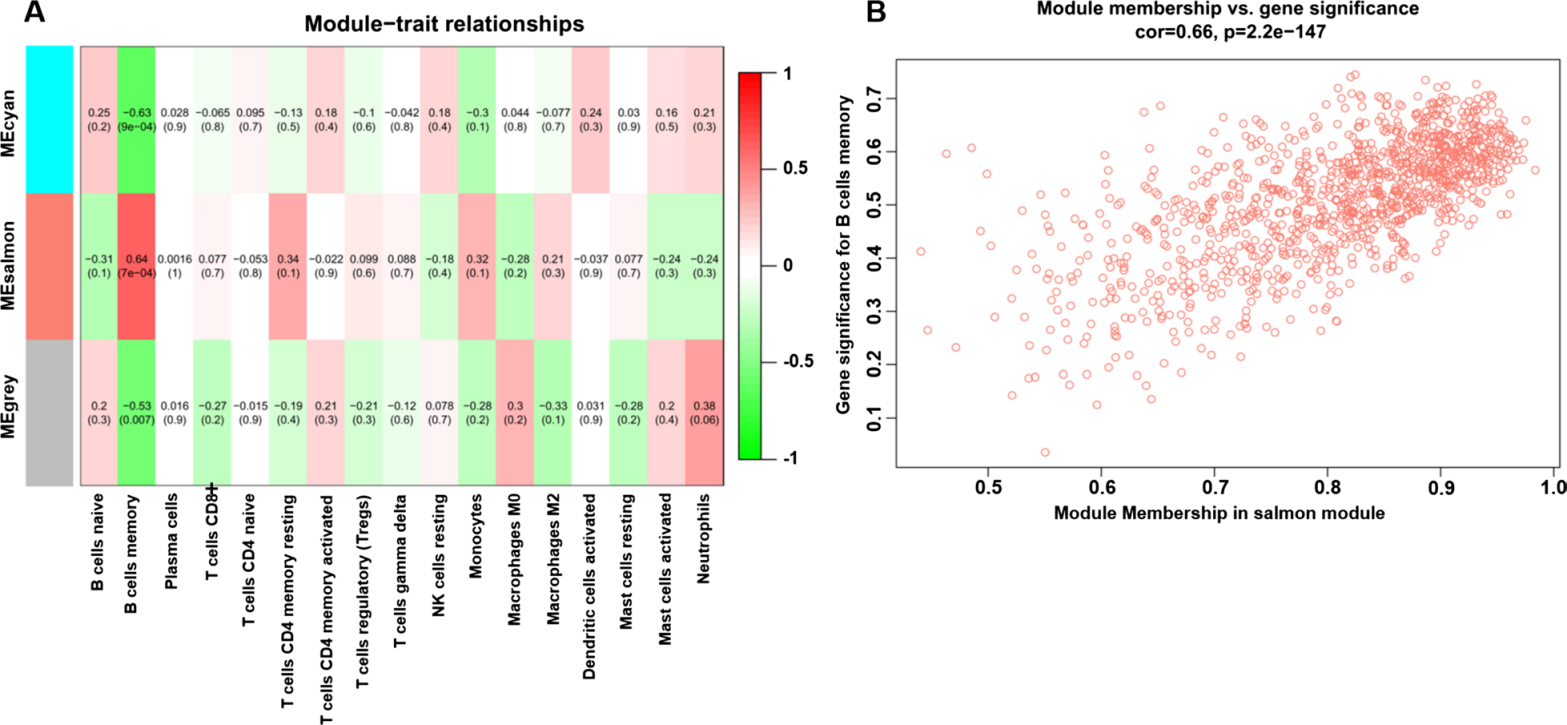
Module-feature associations. **A** Each row corresponds to a modulEigengene and the column to the clinical phenotype. Each cell contains the corresponding correlation in the first line and the *p*-value in the second line. The table is color-coded by correlation according to the color legend. Scatterplot shows a highly significant correlation between gene significant (GS) versus module membership (MM) with memory B cells in the salmon (**B**) module

Based on the expression profile of genes in the midnightblue and lightyellow modules (Additional file 6: Tables S5) and the results of immune cell infiltration in the 24 samples, we identified that the salmon (*r *^*2*^ = 0.64, *p* = 7E-04) module was highly correlated with memory B cells (Fig. 5A). Further in-depth calculations were performed to calculate the correlation coefficient between the colour module and gene significance. Figure 5B demonstrates that the correlation coefficient between the salmon module and gene significance was 0.66 (*p* = 2.2e-147). A total of 1,171 gene symbols in the salmon module and their GS values and corresponding *P* values are also shown in Additional file 7: Tables S6.

### Enrichment analysis of the salmon module

**Fig. 6:**
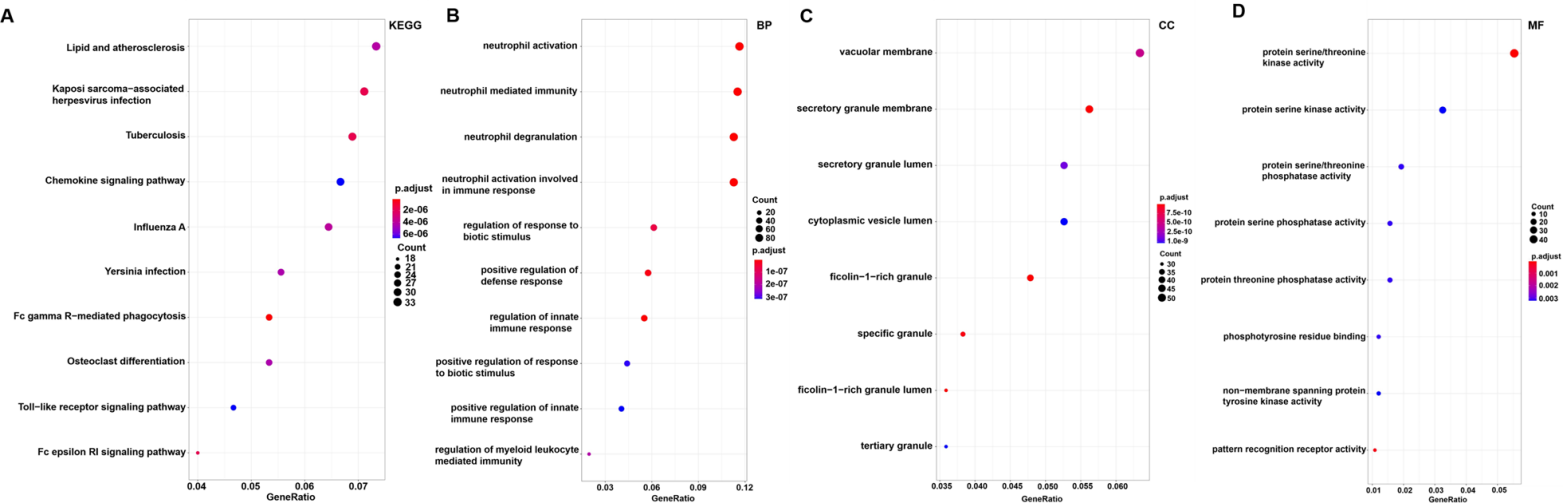
KEGG pathway and GO functional enrichment analyses for genes in the salmon module. The x-axis shows the number of genes and the y-axis shows the KEGG pathway and GO terms. The -log10 (*p*-value) of each term is colored according to the legend. **A** KEGG pathway. **B** Biological process. **C** Cytological components. **D** Molecular function

KEGG pathway and GO functional enrichment analysis of genes in the salmon module were conducted to explore their biological functions. Table 1 and Fig. 6A show the top 10 KEGG pathways. Table 2 shows the results of the GO enrichment analysis, meanwhile Fig. 6B–D show the top 8 biological processes, cellular components, and molecular functions, respectively. The details of these analyses are presented in Additional file 8: Tables S7 and Additional file 9: Table S8.

**Table 1:** KEGG analysis for genes (top 10 significantly enriched terms)

Item	ID	Description	*P*.adjust	geneID
KEGG	hsa04666	Fc gamma R-mediated phagocytosis	3.28E-10	1785/5594/7454/3055/5595/10094/5337/6850/9846/5335/4082/5894/4067/1398/10097/5293/10552/10451/5880/5058/3985/3635/5580/5296
KEGG	hsa05152	Tuberculosis	1.30E-08	4802/5868/5594/3684/7099/5595/51135/5534/26253/533/6850/3460/7097/3459/5993/1378/5894/2033/1051/929/10333/535/1432/3117/7132/818/9114/11151/1509/5603/3126
KEGG	hsa04664	Fc epsilon RI signaling pathway	1.77E-08	5594/241/5595/6850/9846/5335/5894/4067/240/5293/10451/5880/1432/6655/2205/3635/5603/5296
KEGG	hsa05167	Kaposi sarcoma-associated herpesvirus infection	2.21E-08	5594/3055/2783/1147/5595/5534/6850/2787/7311/57580/5335/3459/6774/3454/5894/2033/4067/6233/3661/5293/3455/7297/1432/7132/2793/2932/5603/2790/4792/5296/7538/3133
KEGG	hsa05164	Influenza A	6.29E-08	3836/5594/1147/7099/5595/51135/3460/3459/3454/5894/9021/2033/5611/293/23633/3661/5293/10241/3455/7297/6041/3117/7132/29108/3126/51284/896/4792/5296
KEGG	hsa05417	Lipid and atherosclerosis	8.04E-08	4318/5594/1147/7099/3305/5595/51135/5534/3326/5335/7097/6774/4067/4689/22926/3661/5293/6648/929/10333/10451/6256/1432/7132/818/3310/29108/2932/19/5603/3304/4792/5296
KEGG	hsa05135	Yersinia infection	1.07E-07	5594/7454/1147/7099/5595/10094/51135/391/5335/6195/1398/10097/3661/5293/10552/10451/5880/1432/3678/29108/2932/5603/920/4792/5296
KEGG	hsa04380	Osteoclast differentiation	1.14E-07	126014/5594/1147/5595/5534/6688/6850/11025/9846/3460/3459/7305/3454/9021/4689/5293/3455/7297/1432/7132/5603/7048/4792/5296
KEGG	hsa04620	Toll-like receptor signaling pathway	1.93E-07	5594/1147/51311/7099/5595/51135/7097/3454/7100/3661/5293/54472/3455/929/10333/1432/6696/5603/51284/4792/5296

*KEGG* Kyoto Encyclopedia of Genes and Genome pathway enrichment analyses

**Table 2:** GO analysis for genes (top 8 significantly enriched terms)

Item		ID	Description	GeneRatio	*P*.adjust	geneID
BP	GO:0042119		neutrophil activation	95/816	1.32E-31	8993/55276/978/338339/79930/2548/10970/961/199675/51646/4318/126014/5594/11031/124583/10555/51316/8694/6556/8972/5005/3101/3684/51719/5724/101/25797/27180/5337/391/1992/10533/6850/11025/3326/57580/7097/9545/7305/5023/2268/116844/3482/5236/383/64386/83716/2357/353189/201294/8876/53831/1378/84106/53917/5686/57153/5004/5611/240/6515/410/728/10097/8807/1084/290/51382/54472/929/23200/5265/11010/158747/4126/57126/535/1432/28988/2821/3310/5701/2150/9961/966/51411/1509/29108/3614/10694/8635/2219/5580/3304/7133
BP	GO:0002446		neutrophil mediated immunity	94/816	3.20E-31	8993/55276/978/338339/79930/2548/10970/961/199675/51646/4318/126014/5594/11031/124583/10555/51316/8694/6556/8972/5005/3101/3684/51719/5724/101/25797/27180/51135/5337/391/1992/10533/6850/11025/3326/7097/9545/7305/5023/2268/116844/3482/5236/383/64386/83716/2357/353189/201294/8876/53831/1378/84106/53917/5686/57153/5004/5611/240/6515/410/728/10097/1084/290/51382/54472/929/23200/5265/11010/158747/4126/57126/535/1432/28988/2821/3310/5701/2150/9961/966/51411/1509/29108/3614/10694/8635/2219/5580/3304/7133
BP	GO:0043312		neutrophil degranulation	92/816	6.51E-31	8993/55276/978/338339/79930/2548/10970/961/199675/51646/4318/126014/5594/11031/124583/10555/51316/8694/6556/8972/5005/3101/3684/51719/5724/101/25797/27180/5337/391/1992/10533/6850/11025/3326/7097/9545/7305/5023/2268/116844/3482/5236/383/64386/83716/2357/353189/201294/8876/53831/1378/84106/53917/5686/57153/5004/5611/240/6515/410/728/10097/1084/290/51382/54472/929/23200/5265/11010/158747/4126/57126/535/1432/28988/2821/3310/5701/9961/966/51411/1509/29108/3614/10694/8635/2219/5580/3304/7133
BP	GO:0002283		neutrophil activation involved in immune response	92/816	8.12E-31	8993/55276/978/338339/79930/2548/10970/961/199675/51646/4318/126014/5594/11031/124583/10555/51316/8694/6556/8972/5005/3101/3684/51719/5724/101/25797/27180/5337/391/1992/10533/6850/11025/3326/7097/9545/7305/5023/2268/116844/3482/5236/383/64386/83716/2357/353189/201294/8876/53831/1378/84106/53917/5686/57153/5004/5611/240/6515/410/728/10097/1084/290/51382/54472/929/23200/5265/11010/158747/4126/57126/535/1432/28988/2821/3310/5701/9961/966/51411/1509/29108/3614/10694/8635/2219/5580/3304/7133
BP	GO:0045088		regulation of innate immune response	45/816	1.65E-09	338339/11213/8454/5721/149628/3055/1147/51311/7099/101/26253/6850/4068/3460/3326/3459/7305/2268/383/3148/1378/5894/9021/2033/5686/5699/4067/1398/3661/8807/11126/56339/3455/5591/6041/7294/5058/10623/5701/29108/5691/2219/9252/5580/3133
BP	GO:0031349		positive regulation of defense response	47/816	1.26E-08	338339/961/8454/5721/149628/3055/1147/51311/7099/101/5595/1050/26253/6850/4068/7097/7305/383/3148/5894/2033/5686/5699/4067/1051/3661/8807/11126/5591/63940/7132/7294/5058/10623/5701/2150/29108/5008/64332/5691/5603/2219/9252/5580/51284/4792/3133
BP	GO:0002831		regulation of response to biotic stimulus	50/816	6.38E-08	338339/11213/8454/5721/149628/3055/1147/51311/10269/7099/101/5595/26253/6850/4068/3460/3326/3459/7305/2268/383/3148/1378/5894/9021/2033/5686/5699/4067/1398/3661/10221/8807/11126/56339/3455/5591/6041/7294/5058/10623/5701/2150/29108/5691/2219/9252/5580/81545/3133
BP	GO:0002833		positive regulation of response to biotic stimulus	36/816	3.08E-07	338339/8454/5721/149628/3055/1147/51311/7099/101/26253/6850/4068/7305/383/3148/5894/2033/5686/5699/4067/3661/8807/11126/5591/7294/5058/10623/5701/2150/29108/5691/2219/9252/5580/81545/3133
CC	GO:0101002		ficolin-1-rich granule	40/836	5.86E-15	55276/978/338339/79930/2548/51646/4318/5594/124583/6556/8972/3101/51719/101/25797/10533/3326/116844/5236/83716/2357/1378/5686/240/6515/10097/5265/535/1432/28988/2821/3310/5701/9961/51411/1509/3614/10694/2219/3304
CC	GO:1904813		ficolin-1-rich granule lumen	30/836	1.39E-12	55276/978/51646/4318/5594/124583/3101/51719/25797/10533/3326/116844/5236/83716/5686/240/10097/5265/1432/28988/2821/3310/5701/9961/51411/1509/3614/10694/2219/3304
CC	GO:0030667		secretory granule membrane	47/836	3.16E-12	338339/79930/2548/10970/961/199675/11031/134957/10555/8694/6556/8972/3684/5724/101/27180/5337/391/11025/762/7097/9545/7305/5023/3482/64386/2357/353189/8876/53831/1378/53917/57153/6515/728/1084/290/51382/929/23200/11010/158747/4126/57126/535/966/7133
CC	GO:0042581		specific granule	32/836	2.64E-11	8993/338339/10970/961/199675/126014/124583/10555/8694/5005/3684/101/25797/5337/5023/116844/383/64386/353189/53831/57153/5004/311/6515/1084/51382/54472/158747/57126/966/1509/7133
CC	GO:0005774		vacuolar membrane	53/836	4.34E-10	2548/10970/51296/2629/79901/528/206358/9842/2783/55062/51311/5337/533/89849/9516/7311/3326/9545/523/58528/8408/2357/353189/9043/8876/6272/57153/51310/7805/11342/526/7056/10241/290/51382/23200/11010/10211/23531/4126/535/3117/7942/1175/4864/219931/9528/9114/1509/28962/9583/3126/51284
CC	GO:0034774		secretory granule lumen	44/836	7.37E-10	8993/55276/978/2153/126014/5594/124583/51316/5005/3101/51719/25797/1992/10533/3326/2268/116844/383/83716/201294/5686/5004/5611/240/410/10097/54472/81/5265/3959/1432/28988/2821/3310/5701/9961/51411/1509/29108/3614/10694/8635/2219/5580
CC	GO:0070820		tertiary granule	30/836	8.41E-10	8993/978/338339/79930/2548/961/199675/51646/4318/126014/124583/6556/8972/3684/5724/101/25797/5337/116844/5236/2357/53831/1378/5004/6515/57126/535/28988/966/1509
CC	GO:0060205		cytoplasmic vesicle lumen	44/836	8.41E-10	8993/55276/978/2153/126014/5594/124583/51316/5005/3101/51719/25797/1992/10533/3326/2268/116844/383/83716/201294/5686/5004/5611/240/410/10097/54472/81/5265/3959/1432/28988/2821/3310/5701/9961/51411/1509/29108/3614/10694/8635/2219/5580
MF	GO:0004674		protein serine/threonine kinase activity	46/831	5.86E-15	55276/978/338339/79930/2548/51646/4318/5594/124583/6556/8972/3101/51719/101/25797/10533/3326/116844/5236/83716/2357/1378/5686/240/6515/10097/5265/535/1432/28988/2821/3310/5701/9961/51411/1509/3614/10694/2219/3304
MF	GO:0038187		pattern recognition receptor activity	9/831	1.39E-12	55276/978/51646/4318/5594/124583/3101/51719/25797/10533/3326/116844/5236/83716/5686/240/10097/5265/1432/28988/2821/3310/5701/9961/51411/1509/3614/10694/2219/3304
MF	GO:0004722		protein serine/threonine phosphatase activity	16/831	3.16E-12	338339/79930/2548/10970/961/199675/11031/134957/10555/8694/6556/8972/3684/5724/101/27180/5337/391/11025/762/7097/9545/7305/5023/3482/64386/2357/353189/8876/53831/1378/53917/57153/6515/728/1084/290/51382/929/23200/11010/158747/4126/57126/535/966/7133
MF	GO:0106306		protein serine phosphatase activity	13/831	2.64E-11	8993/338339/10970/961/199675/126014/124583/10555/8694/5005/3684/101/25797/5337/5023/116844/383/64386/353189/53831/57153/5004/311/6515/1084/51382/54472/158747/57126/966/1509/7133
MF	GO:0106307		protein threonine phosphatase activity	13/831	4.34E-10	2548/10970/51296/2629/79901/528/206358/9842/2783/55062/51311/5337/533/89849/9516/7311/3326/9545/523/58528/8408/2357/353189/9043/8876/6272/57153/51310/7805/11342/526/7056/10241/290/51382/23200/11010/10211/23531/4126/535/3117/7942/1175/4864/219931/9528/9114/1509/28962/9583/3126/51284
MF	GO:0001784		phosphotyrosine residue binding	10/831	7.37E-10	8993/55276/978/2153/126014/5594/124583/51316/5005/3101/51719/25797/1992/10533/3326/2268/116844/383/83716/201294/5686/5004/5611/240/410/10097/54472/81/5265/3959/1432/28988/2821/3310/5701/9961/51411/1509/29108/3614/10694/8635/2219/5580
MF	GO:0106310		protein serine kinase activity	27/831	8.41E-10	8993/978/338339/79930/2548/961/199675/51646/4318/126014/124583/6556/8972/3684/5724/101/25797/5337/116844/5236/2357/53831/1378/5004/6515/57126/535/28988/966/1509
MF	GO:0004715		non-membrane spanning protein tyrosine kinase activity	10/831	8.41E-10	8993/55276/978/2153/126014/5594/124583/51316/5005/3101/51719/25797/1992/10533/3326/2268/116844/383/83716/201294/5686/5004/5611/240/410/10097/54472/81/5265/3959/1432/28988/2821/3310/5701/9961/51411/1509/29108/3614/10694/8635/2219/5580

*BP* biological processes, *CC* cellular components, *MF* molecular functions

### Construction of the PPI network and identification of hub-genes

**Fig. 7:**
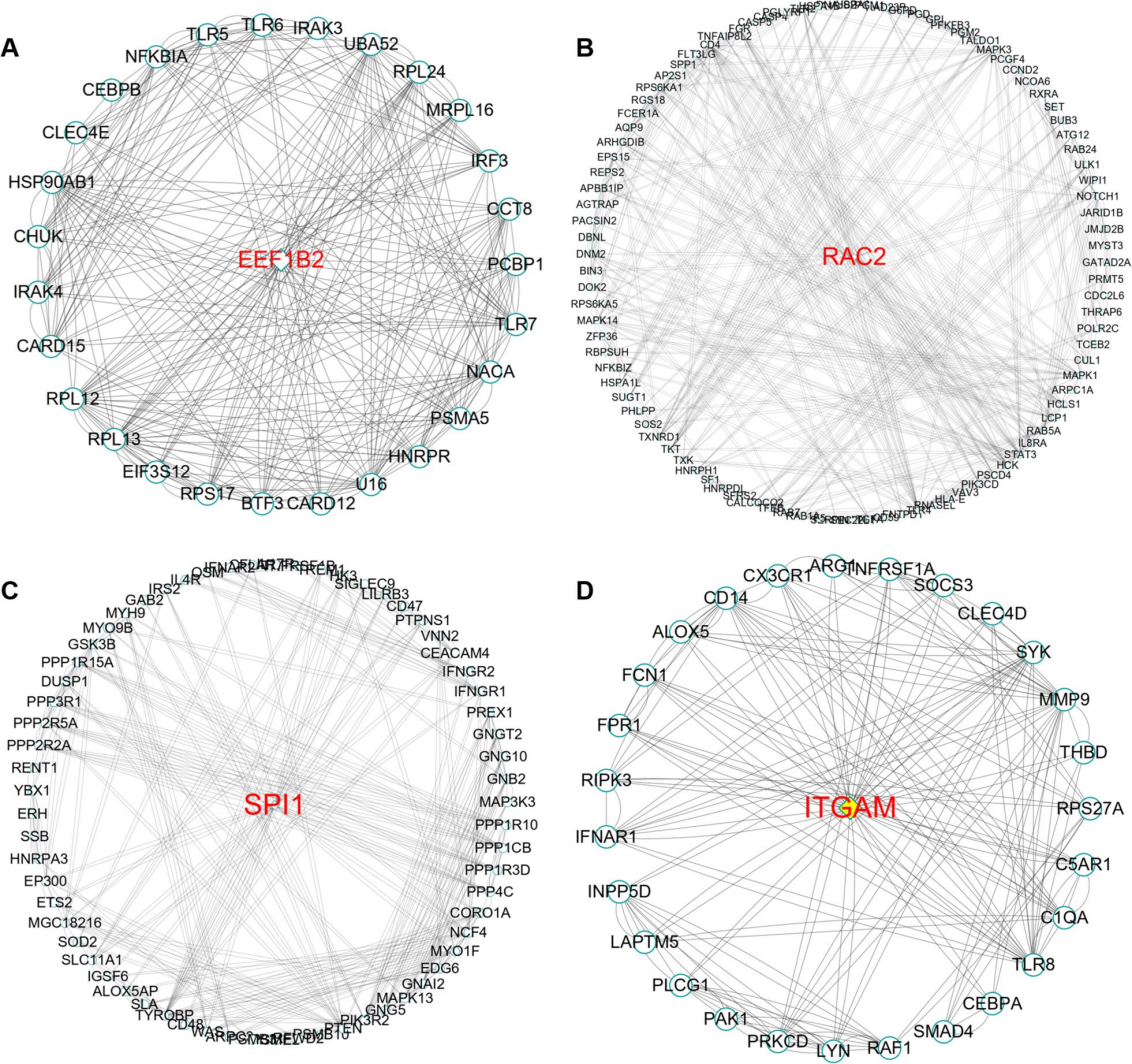
MCODE analysis based on PPI network. **A** Module-1 with MCODE score = 10.44. **B** Module-2 with MCODE score = 7.306. **C** Module-3 with MCODE score = 6.827. **D** Module-4 with MCODE score = 6.263

As shown in Additional file 1: Figure S1, a PPI network with 1,088 nodes and 5,960 edges was built using the STRING tool. The MCODE plug-in in Cytohubba software was used to analyse the PPI network. Module-1 (Fig. 7A) had a score of 10.44, the module-2 (Fig. 7B) score was 7.306, module-3 (Fig. 7C) score was 6.827, while module-4 (Fig. 7D) score was 6.263. In addition, the eukaryotic translation elongation factor 1 beta 2 (*EEF1B2*) with a degree of 30 in module-1, the Rac family small GTPase 2 (*RAC2*) with a degree of 46 in module-2, *SPI1* with a degree of 38 in module-3, and *ITGAM* with a degree of 40 in module-4 were identified as hub genes closely associated with AMI.

### The correlation between key genes and immune cells

**Fig. 8:**
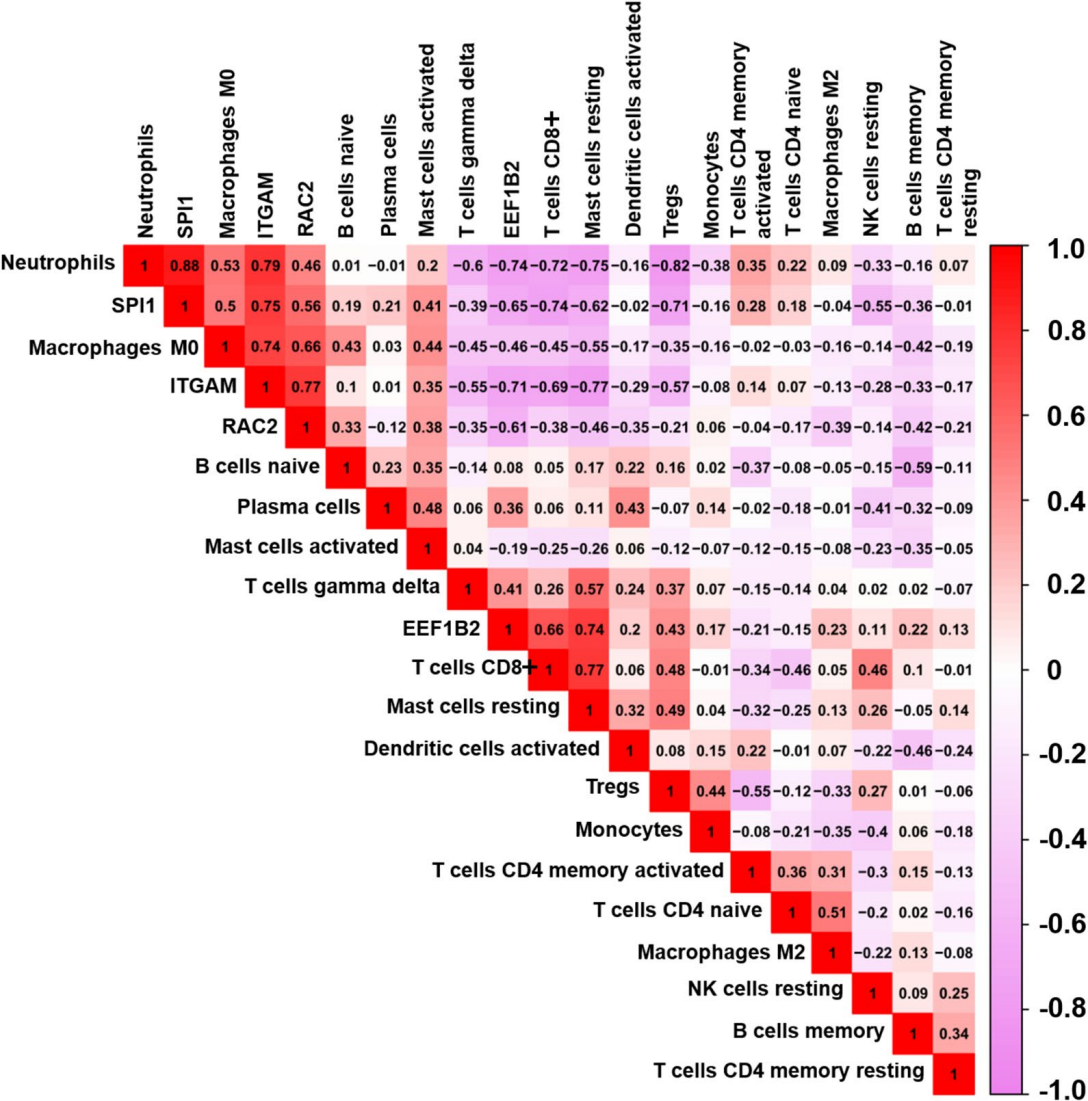
The correlation between key genes and immune cells

As shown in Fig. 8, a correlation matrix was used to determine the correlation between key genes and immune cells. *EEF1B2* was found to be positively correlated with γδ T cells, CD8 + T cells, Tregs, and resting mast cells, but negatively correlated with neutrophils and M0 macrophages. *RAC2* was negatively correlated with γδ T cells, CD8 + T cells and resting mast cells, while *SPI1* and *ITGAM* were positively correlated with neutrophils and M0 macrophages but negatively correlated with γδ T cells, CD8 + T cells, resting mast cells, and Tregs.

### RT-qPCR

**Fig. 9:**
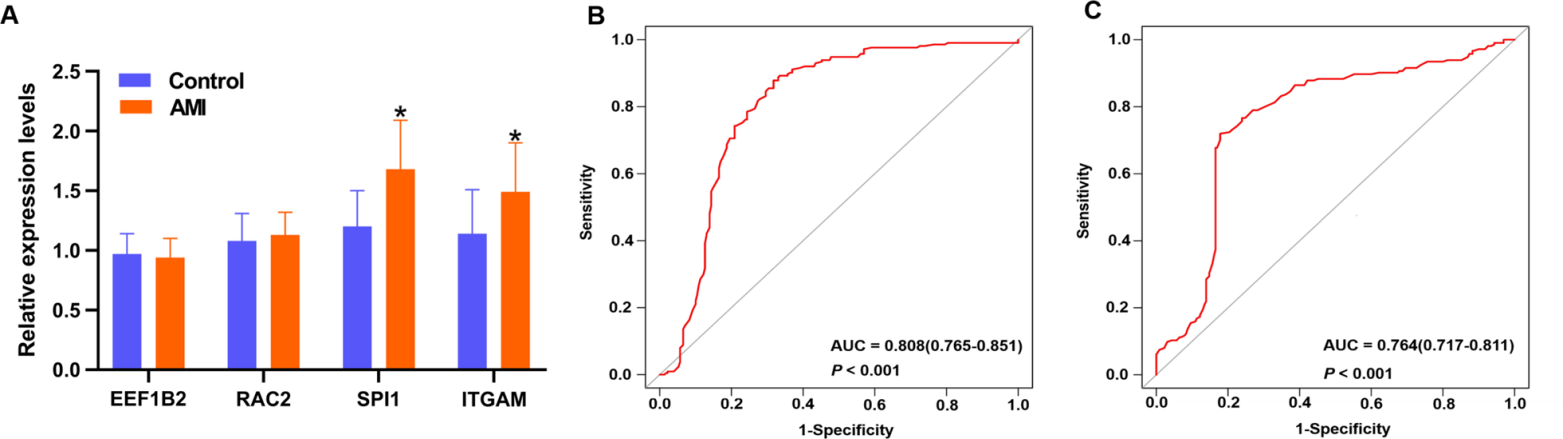
qRT-PCR validation and the ROC curves analysis. **A** The relative expression levels of *EEF1B2*, *RAC2*, *SPI1* and *ITGAM*. The AUC value of *SPI1* **B** was 0.808 (95% CI 0.765–0.851; *P* < 0.001) and *ITGAM* **C** was 0.764 (95% CI 0.717–0.811; *P* < 0.001) for prediction of AMI risk

The results of the RT-qPCR indicated that the expression levels of *SPI1* and *ITGAM* were significantly elevated in AMI patients compared with controls (Fig. 9A).

### ROC curve for AMI patients

As shown in Fig. 9B, C, the ROC curve analysis was used to calculate the predictive values of *SPI1* and *ITGAM* for AMI patients. The AUC values of *SPI1* and *ITGAM* were 0.808 (95% CI 0.765–0.851; *p* < 0.001) and 0.764 (95% CI 0.717–0.811; *p* < 0.001) for the prediction of AMI risk, respectively.

### Demographic and biochemical characteristics

Several clinical features have no significant differences between AMI patients and controls, including heart rate, age, diastolic blood pressure, sex ratio, apolipoprotein (Apo) B, height and the proportion of alcohol consumed (Table 3). However, compared with controls, AMI cases had a higher proportion of smoke, and had higher pulse pressure, uric acid, glucose levels, systolic blood pressures, levels of triglyceride (TG), LDL-C and TC levels, weight, cardiac troponin T (cTnT) levels, creatinine levels, creatine kinase (CK), CK-MB and body mass index (BMI). Moreover, the levels of serum ApoA1, high-density lipoprotein cholesterol (HDL-C), and the ApoA1/ApoB ratio were remarkably higher in controls than in AMI patients.

**Table 3:** Comparison of demographic, lifestyle characteristics and serum lipid levels of the participants

Characteristic	Control(n = 214)	AMI (n = 230)	Test‑statistic	*P*
Male/female ^c^	158/56	169/61	0.007	0.933
Age (years)^a^	57.51 ± 11.07	58.98 ± 11.87	0.002	0.195
Height (cm)^a^	162.68 ± 7.64	163.30 ± 7.82	1.110	0.394
Weight (kg)^a^	56.72 ± 8.17	62.62 ± 11.60	28.115	< 0.001
BMI (kg/m^2^)^a^	20.93 ± 3.09	23.39 ± 3.50	1.824	< 0.001
Smoking [n (%)]^c^	50(32.9)	79 (41.5)	6.488	0.011
Alcohol [n (%)]^c^	57(26.7)	67(26.2)	0.343	0.558
SBP (mmHg)^a^	121.51 ± 15.61	136.77 ± 20.34	24.528	< 0.001
DBP (mmHg)^a^	76.36 ± 9.32	77.85 ± 11.90	8.232	0.145
PP (mmHg)^a^	45.15 ± 11.10	58.92 ± 19.47	59.144	< 0.001
Glu (mmol/L)^a^	6.05 ± 1.57	6.45 ± 1.76	8.646	0.009
TC (mmol/L)^a^	4.36 ± 0.80	4.62 ± 0.92	6.716	0.002
TG (mmol/L)^b^	0.88(0.43)	1.23(0.66)	-7.141	< 0.001
HDL-C (mmol/L)^a^	1.82 ± 0.45	1.24 ± 0.36	9.799	< 0.001
LDL-C (mmol/L)^a^	2.68 ± 0.68	3.14 ± 0.96	14.149	< 0.001
ApoA1 (g/L)^a^	1.41 ± 0.28	1.01 ± 0.23	1.654	< 0.001
ApoB (g/L)^a^	0.85 ± 0.18	0.84 ± 0.25	10.485	0.803
ApoA1/ApoB^a^	1.72 ± 0.46	1.30 ± 0.50	0.449	< 0.001
Heart rate (beats/minutes)^a^	73.08 ± 9.76	73.53 ± 7.61	7.097	0.582
Creatinine, (μmol/L)^a^	70.72 ± 11.39	74.32 ± 12.64	1.043	0.002
Uric acid, (μmol/L)^a^	258.57 ± 70.12	274.89 ± 80.99	7.625	0.024
Troponin T, (μg/L)^a^	0.06 ± 0.03	3.56 ± 1.90	216.138	< 0.001
CK, (U/L)^a^	72.40 ± 40.58	1055.62 ± 538.35	271.429	< 0.001
CK-MB, (U/L)^a^	12.11 ± 3.11	133.41 ± 37.74	824.115	< 0.001

*SBP* Systolic blood pressure, *DBP* Diastolic blood pressure, *PP* Pulse pressure, *Glu* Glucose, *HDL-C* high-density lipoprotein cholesterol, *LDL-C* low-density lipoprotein cholesterol, *Apo* Apolipoprotein, *TC* Total cholesterol,* TG* Triglyceride

^a^Continuous data were presented as means ± SD and determined by two side *t*-test

^b^A chi-square analysis was used to evaluate the difference of the rate between the groups

## Discussion

In the past, the formation of atherosclerotic plaque as a result of dyslipidemia it was considered as a major cause of arteriosclerosis. However, during recent years further research has shown that arteriosclerosis is actually a chronic inflammatory process that induces strong immune activity [28]. Previous studies have suggested that a variety of immune cells play a key part in atherosclerosis. Dounousi et al. suggested that monocyte subsets play a crucial role in atherogenesis and inflammatory cascades in cardiovascular disease. Increasing counts and activity of monocytes are closely related to the clinical indexes of chronic kidney disease (CKD), atherosclerosis, and heart failure [29]. T lymphocytes are the most critical immune cells found in vivo. Based on their surface markers and functions, T lymphocytes can be classified as CD4 + and CD8 + cell subgroups. CD8 + T cells play a dual role in atherosclerosis. A compelling study pointed out that CD8 + T cells could secrete a variety of inflammatory cytokines, which could aggravate the inflammatory response and increase the instability of atherosclerotic plaques [30]. Inversely, cytotoxic activity that targets antigen presenting cells and regulatory CD8 + T cell subsets could effectively suppressed the progression of atherosclerosis by alleviating the immune reaction [30]. Other immune cell types, including neutrophils [31] and mast cells [32], also play a key part in the progression of cardiovascular disease. Notably, Han et al. suggested that the proportion of activated dendritic cells and Tfhs in CAD was remarkably higher and that the proportion of Tregs, resting CD4 + T cells, and γδ T cells was remarkably lower than in the control group. In addition, Yang et al. also identified an increase in the infiltration of monocytes but a decrease in the infiltration of CD8 + T cells in CAD subjects [10]. This data indicates that CAD exhibits inflammatory microenvironment patterns. On the contrary, persistent T-cell responses induced by myocardial infarction are significantly correlated with subsequent left ventricular remodelling, which ultimately leads to cardiac arrest and heart failure [33]. These results indicate that the immune system plays a very complex role in the pathophysiology of CAD. However, the pattern of immune cell infiltration in AMI has not been fully elucidated. To further explore the proportions and types of immune cells in AMI patients, the CIBERSORT package of R software was used to conduct a comprehensive assessment of 22 types of immune cell infiltration in AMI cases. We noticed that there was a decrease in the infiltration of CD8 + T cells, resting mast cells, and γδ T cells but an increase in the infiltration of neutrophils and M0 macrophages in AMI patients. These results indicate that there may be a difference in the immune cell infiltration pattern between AMI and CAD. These differences can better help us understand which immune cells play a vital part in processes from the deterioration of CAD to AMI. As previously mentioned, compared with normal samples, the proportion of neutrophils, which are involved in ischemic injury after stroke in ischemic stroke (IS) samples is generally higher. Neutrophils may be a promising target for IS therapies [34]. In addition, CD8 + T cells have pleural effects on atherosclerosis, and our study showed that the proportion of neutrophils were higher and that the proportion of CD8 + T cells were lower in the AMI group than in the control group. This indicates that neutrophils can accelerate but CD8 + T cells inhibit the occurrence and progression of AMI. Nevertheless, it is not clear whether the number of CD8 + T cells and neutrophils in peripheral blood samples reflect their infiltration into the vascular wall. Additionally, the current study also revealed that there were several different interactions between different immune cells in AMI. We noticed that neutrophils were negatively related to Tregs, CD8 + T cells, γδ T cells, and resting mast cells, while CD8 T + cells were positively related to resting mast cells. The immune cells infiltration analysis suggested a complicated network in AMI. Nevertheless, the potential mechanism of these relationship between infiltrated immune cells needs to be verified using in vivo and in vitro studies.

To further identify immune-related key genes involved in AMI, we conducted WGCNA combined with CIBERSORT to screen key modules that were remarkably associated with immune cells, and it was indicated that the salmon module was remarkably related to the memory B cells. Then, KEGG and GO enrichment analyses were conducted to further confirm that the genes in the salmon module were mainly involved in immune related signalling pathways and biological processes. A PPI network was built based on genes in the salmon module. Following the MCODE analysis, four different MCODE complexes were identified in the salmon module, and four hub genes (*EEF1B2*, *RAC2*, *SPI1* and *ITGAM*) that were significantly correlated with AMI were identified. External validation showed that the expression levels of *ITGAM* and *SPI1* were significantly different between AMI and the control group, while the expression levels of the *EEF1B2* and *RAC2* genes were not significantly different between the two groups. These results suggest that *ITGAM* and *SPI1* genes may act as key immune-related genes involved in AMI.

Previous research has revealed that *ITGAM* is a member of the β2 integrin family of adhesion molecules, and adhesion molecules play an indispensable role in the recruitment and activation of neutrophils, macrophages, and monocytes during the process of inflammation [35]. Zirlik et al. proved that *ITGAM* plays a key role in inflammatory processes, such as the neutrophils and monocytes adhesion to injured endothelial cells and trans-endothelial migration, and is also involved in CD40 ligand-mediated atherosclerotic inflammation [36]. Previous studies have shown that the transcription profiles of monocytes following AMI in mice and human share common biological characteristics. *ITGAM* is one of the most common inflammation-related genes, has been shown to play a key role in monocyte inflammation, intercellular signal transduction, and cell proliferation [37]. On the other hand, Wang et al. found that *ITGAM* expression was correlated with various immune cells, including Tregs, M2 Macrophages, and that *ITGAM* plays an important role in acute myeloid leukaemia (AML) related immune regulation. Elevated *ITGAM* expression levels could predict poor overall survival and poor initial treatment response in patients with AML [38]. In addition, Ayari et al. found that *ITGAM* was significantly overexpressed in human carotid plaque [39]. Similarly, in our previous study, it was found that the expression levels of *ITGAM* were significantly upregulated in patients with CAD, and that high expression levels of *ITGAM* showed high diagnostic efficiency for the recognition ability of CAD [40]. However, to our knowledge, no reports have been published on the relationship between *ITGAM* and the immune microenvironment in AMI. In the current study, we noticed that *ITGAM* is positively correlated with neutrophils and negatively correlated with CD8 + T cells and resting mast cells. Meanwhile, we also noted that *ITGAM* was significantly overexpressed in patients with AMI. Based on these results, we speculated that the level of *ITGAM* overexpression is significantly correlated with the occurrence and development of AMI, and that *ITGAM* is expected to be a novel immune-related target for the prevention and treatment of AMI.

S*PI1* encodes an ETS-domain transcription factor, PU.1, which is essential for the development of myeloid cells and B lymphocytes, and is the primary regulator of cell-to-cell communication in the immune system [41]. Pulugulla et al. noticed that the expression level of *SPI1* mRNA is upregulated in activated T cells, and it may play a role in regulating the expression of interleukin 1 beta (*IL1B*) following the activation of CD4 T cells [42]. Yashiro et al. found that *SPI1* could activate the C–C motif chemokine ligand 22 (*CCL22*) gene in dendritic cells and macrophages by directly binding to two key elements in the promoter, thereby mediating the migration of different subsets of leukocytes during the immune response [43]. The continuous overexpression of *SPI1* in hematopoietic cells leads to the differentiation of macrophages, and *SPI1* is an important regulatory factor for all states of tumour-associated macrophages (TAMs). Inhibition of the expression of *SPI1* can effectively reduce the maturation and polarization of TAMs to play an anti-tumour role [44]. Previous studies have suggested that *SPI1* acts as a key transcription factor that regulates the expression of several inflammatory genes, and has been found to be significantly overexpressed in advanced plaques. High expression levels of *SPI1* showed modest efficiency in distinguishing the capacity of CAD [45]. Similarly, Qiao found that *SPI1* plays a key role in the occurrence and development of ischemic cardiomyopathy and dilated cardiomyopathy by regulating apoptosis- and inflammation-related genes [46]. In addition, *SPI1* has been predicted to regulate the expression of key genes that lead to heart failure following AMI [47]. However, the correlation between *SPI1* and the immune microenvironment of AMI has not been reported on. Fortunately, in this research study, we noted that *SPI1* is positively correlated with *ITGAM* and neutrophils but negatively correlated with Tregs, CD8 + T cells, and resting mast cells. Meanwhile, the gene expression level of *SPI1* in AMI patients was also significantly higher than those in the control group. This suggests that *SPI1* may be a novel potential molecular target for the diagnosis and treatment of AMI.

The current research study has several limitations. First, the validation samples included in this study were recruited from a single centre and had a small sample size. It is not clear whether the findings of this study are similar among individuals in other regions and ethnic groups. Therefore, the validity of the results of this study need to be further tested using multi-centre and larger samples. Second, it is not clear whether *SPI1* acts as a transcription factor to regulate the expression of *ITGAM*. Third, further in vivo and in vitro research is needed to clarify the underlying mechanism of the correlations between *ITGAM* and *SPI1*expression levels and the infiltration of immune cells in AMI.

## Conclusions

Immune cell infiltration plays a crucial role in the occurrence and development of AMI. *ITGAM* and *SPI1* are the key immune-related genes that have the potential to become targets for the prevention and treatment of AMI.

## Supplementary Information

**Additional file 1: Figure S1**. PPI network of genes in salmon module. The edge shows the interaction between two genes.

**Additional file 2: Table S1**. The expression profile of the 24,958 genes in the GSE61144 dataset.

**Additional file 3: Table S2**. Tables S2 The clinical features of the 24 samples in the GSE61144 dataset.

**Additional file 4: Table S3**. Tables S3 The GS values as well as corresponding p values of 2993 genes in the midnightblue and lightyellow modules.

**Additional file 5: Table S4**. The immune cell infiltration pattern of AMI samples in the GSE61144 dataset.

**Additional file 6: Table S5**. The expression profile of 2993 genes in the midnightblue and lightyellow modules.

**Additional file 7: Table S6**. The GS values as well as corresponding p values of 1171 genes in the salmon module.

**Additional file 8: Table S7**. Detailed results of the KEGG enrichment analysis.

**Additional file 9: Table S8**. Detailed results of the GO enrichment analysis.

## Data Availability

The raw data supporting the conclusions of this article will be made available by the authors, without undue reservation.

## Abbreviations

WGCNA: Weighted gene co-expression network analysis
CAD: Coronary artery disease
GS: Gene significance
AMI: Acute myocardial infarction
γδ: Gamma beta
Tregs: Regulatory T cells
NO: Nitric oxide
TNF-α: Tumor necrosis factor-alpha
IL: Interleukin
CRP: C-reactive protein
MACEs: Major adverse cardiovascular events
DAVID: Database for annotation, visualization and integrated discovery
T2DM: Type 2 diabetes mellitus
GO: Gene Ontology
HDL-C: High-density lipoprotein cholesterol
IS: Ischemic stroke
KEGG: Kyoto Encyclopedia of Genes and genomes
LDL-C: Low-density lipoprotein cholesterol
MCODE: Molecular Complex Detection
Apo: Apolipoprotein
PPI: Protein–protein interaction
GEO: Gene Expression Omnibus
BMI: Body mass index
TG: Triglyceride
RT-qPCR: Quantitative real time polymerase chain reaction
TC: Total cholesterol
PCI: Percutaneous coronary intervention
ECG: Electrocardiogram
TOM: Topological overlap matrix
Mes: Module eigengenes
MM: Module membership
STRING: Search Tool for the Retrieval of Interacting Genes
CK-MB: Creatine kinase-MB
cTns: Cardiac troponins
cTnT: Cardiac troponin T
ITGAM: Integrin subunit alpha M
CVD: Cardiovascular diseases
Mes: Module eigengenes
CKD: Chronic kidney disease
BMI: Body mass index
CK: Creatine kinase
IS: Ischemic stroke
AML: Acute myeloid leukemia
LRRC18: Leucine rich repeat containing 18
ASCC2: Activating signal cointegrator 1 complex subunit 2
SLC25A37: Solute carrier family 25 member 37
PBMCs: Peripheral blood monocytes
SPI1: Spi-1 proto-oncogene
AUCs: Areas under the curves
Tfhs: Follicular helper T cells
IL1B: Interleukin 1 beta
CCL22: C–C motif chemokine ligand 22
TAMs: Tumor-associated macrophages
RAC2: Rac family small GTPase 2
EEF1B2: Eukaryotic translation elongation factor 1 beta 2
