# Supplementary material for: Identifying patterns of immune related cells and genes in the peripheral blood of acute myocardial infarction patients using a small cohort
Source: J Transl Med. 2022 Jul 21;20:321. doi: 10.1186/s12967-022-03517-1 (PMC9306178; doi:10.1186/s12967-022-03517-1)
Supplement: Supplementary file 1 — Additional file 1: Figure S1. PPI network of genes in salmon module. The edge shows the interaction between two genes. [file 12967_2022_3517_MOESM1_ESM.docx]

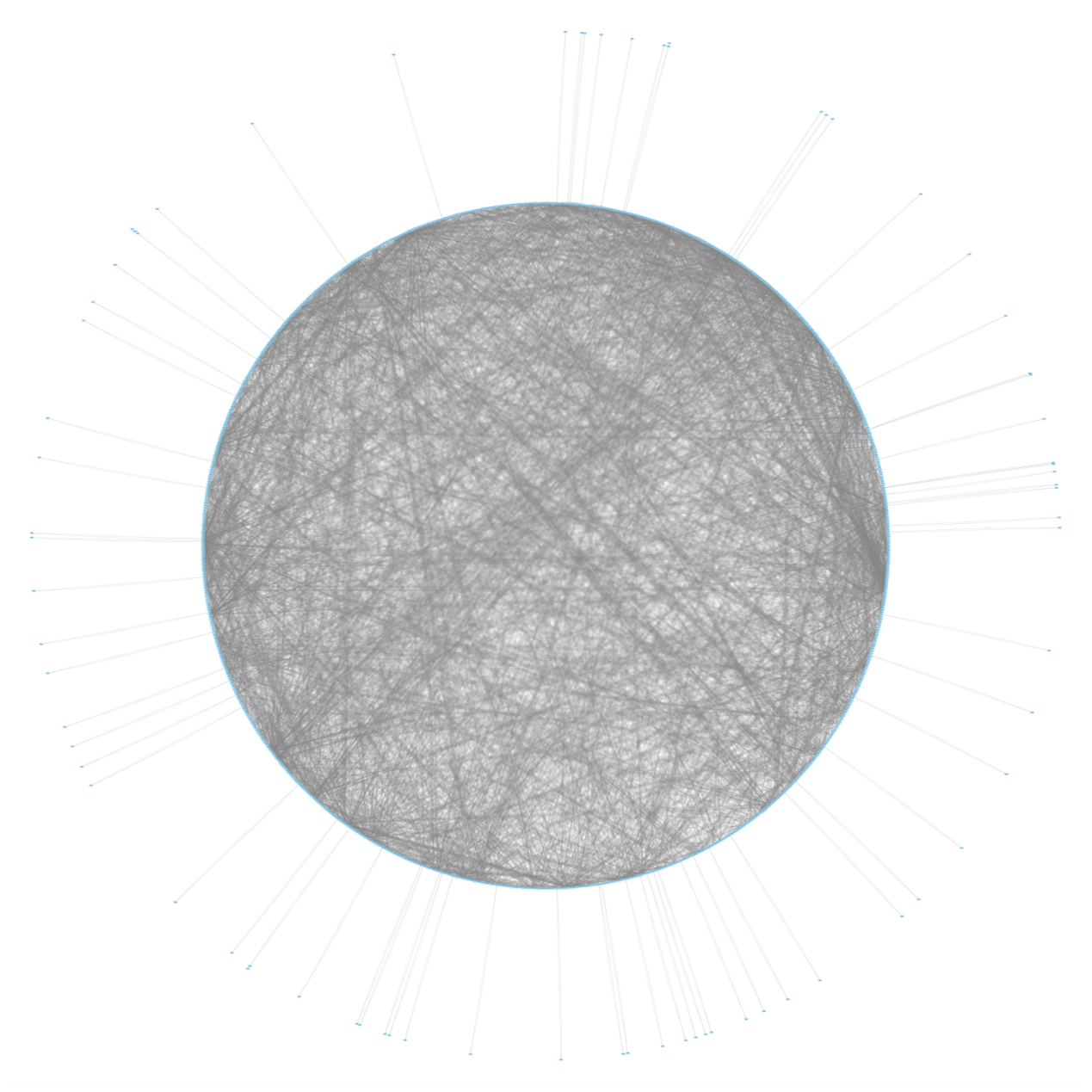


**Additional Figure 1**: PPI network of genes in salmon module. The edge shows the interaction between two genes.
